# Prediction of Malignant Transformation of WHO II Astrocytoma Using Mathematical Models Incorporating Apparent Diffusion Coefficient and Contrast Enhancement

**DOI:** 10.3389/fonc.2021.744827

**Published:** 2021-09-29

**Authors:** Alex Mun-Ching Wong, Tiing Yee Siow, Kuo-Chen Wei, Pin-Yuan Chen, Cheng Hong Toh, Mauricio Castillo

**Affiliations:** ^1^ Department of Medical Imaging and Intervention, Chang Gung Memorial Hospital at Keelung, Keelong, Taiwan; ^2^ College of Medicine, Chang Gung University, Tao‐Yuan, Taiwan; ^3^ Department of Medical Imaging and Intervention, Chang Gung Memorial Hospital at Linkou, Tao‐Yuan, Taiwan; ^4^ Department of Neurosurgery, Chang Gung Memorial Hospital at Linkou, Taoyuan, Taiwan; ^5^ Department of Radiology, University of North Carolina School of Medicine, Chapel Hill, NC, United States

**Keywords:** malignant transformation, contrast enhancement, apparent diffusion coefficient, model prediction and validation, low-grade gliomas

## Abstract

Using only increasing contrast enhancement as a marker of malignant transformation (MT) in gliomas has low specificity and may affect interpretation of clinical outcomes. Therefore we developed a mathematical model to predict MT of low-grade gliomas (LGGs) by considering areas of reduced apparent diffusion coefficient (ADC) with increased contrast enhancement. Patients with contrast-enhancing LGGs who had contemporaneous ADC and histopathology were retrospectively analyzed. Multiple clinical factors and imaging factors (contrast-enhancement size, whole-tumor size, and ADC) were assessed for association with MT. Patients were split into training and validation groups for the development of a predictive model using logistic regression which was assessed with receiver operating characteristic analysis. Among 132 patients, (median age 46.5 years), 106 patients (64 MT) were assigned to the training group and 26 (20 MT) to the validation group. The predictive model comprised age (*P* = 0.110), radiotherapy (*P* = 0.168), contrast-enhancement size (*P* = 0.015), and ADC (*P* < 0.001). The predictive model (area-under-the-curve [AUC] 0.87) outperformed ADC (AUC 0.85) and contrast-enhancement size (AUC 0.67). The model had an accuracy of 84% for the training group and 85% respectively for the validation group. Our model incorporating ADC and contrast-enhancement size predicted MT in contrast-enhancing LGGs.

## Introduction

Malignant transformation (MT) of low-grade gliomas (LGGs) is the histopathologic progression of grade II World Health Organization (WHO) tumors to WHO grade III or IV tumors. LGGs account for 14.6% of gliomas in population-based studies (1) and may remain stable clinically and by imaging for years after initial diagnosis and treatment. MRI features suggesting disease progression include enlargement of non-enhancing areas and increasing enhancement on post-gadolinium T1-weighted images. However, tumors showing these features may remain WHO grade II or undergo MT and correct diagnosis requires histopathologic confirmation. Because of potential risks and costs associated with histopathologic confirmation, increased contrast enhancement is frequently used as a surrogate marker for MT in clinical practice, research studies, and clinical trials of LGG. In the widely used Response Assessment in Neuro-Oncology criteria (2), an increase of enhancement is regarded as MT.

In a recent study, the specificity of using increased contrast enhancement to detect MT was 57%, despite a sensitivity of 92% (3). Among LGGs with increasing contrast enhancement, the percentage of tumors that remained grade II ranges from 18-37% (3, 4). Also, increasing contrast enhancement may be associated with treatment-related changes (5, 6). This limited accuracy of using increasing contrast enhancement to diagnose MT was recognized in a consensus article recently published by the Society for Neuro-Oncology and the European Association of Neuro-Oncology (7). Therefore, using increasing contrast enhancement as a marker of MT may result in overtreatment of patients whose tumors remain low-grade, errors in the results of research studies, and misinterpretation of clinical benefits of new therapies. Because of the issues associated with increasing contrast enhancement, it is crucial to search for imaging markers that can accurately diagnose MT.

Diffusion-weighted imaging (DWI) identifies high-grade gliomas by their low apparent diffusion coefficient (ADC) values (8, 9). In a study investigating multiple diffusion tensor imaging parameters, ADC showed the highest diagnostic performance in differentiating between LGGs and high-grade gliomas (9). A recent study demonstrated the utility of DWI in predicting MT of LGGs (10). Since ADC values can be heterogeneous in previously treated LGGs (11), the choice of regions for ADC measurements affects its reproducibility and accuracy in diagnosing MT. To the best of our knowledge, the role of DWI in diagnosing MT has not been investigated among LGGs with increased contrast enhancement. In our study which used histopathology as the gold standard, we aimed to develop a regression model based on clinical and imaging factors to predict MT in a group of patients with LGGs with increased contrast enhancement on follow-up MRI studies.

## Materials and Methods

### Patients

This retrospective study was performed after institutional review board approval. The need to obtain patient informed consent was waived by our review board (202100387B0). Patients were selected from our brain tumor database if they met the following criteria: 1) prior pathologic diagnosis of LGG and follow-up MRI studies performed between 2004 and 2020 showing increasing contrast enhancement; 2) having undergone surgery due to increased contrast enhancement with the resected tumor being grade II, III, or IV gliomas; 3) availability of DWI, and 4) removal of tumor regions with increased contrast enhancement confirmed by follow-up MRI. Increasing contrast enhancement was defined as new enhancement in previously non-enhancing regions, new separate lesions with contrast enhancement, or at least a 25% increase in the size of enhancement in previously enhancing regions at baseline. Baseline was the first follow-up MRI study after surgery. Increased contrast enhancement was confirmed by neuroradiologists who compared the baseline MRI and the most recent MRI before the next surgery. Patients with multiple surgeries for separate events of increasing contrast enhancement were included until they experienced MT. Each instance of increasing contrast enhancement was treated independently. Histopathologic diagnosis was made by a board-certified neuropathologist according to the 2000 WHO classification of CNS tumors before 2007, the 2007 WHO classification from 2007 to 2016, and the 2016 WHO classification after 2016. We excluded patients younger than 18 years of age at initial diagnosis, those with a diagnosis of radiation necrosis, and those in whom DWI showed susceptibility artifacts that hindered interpretation.

**Figure 1:**
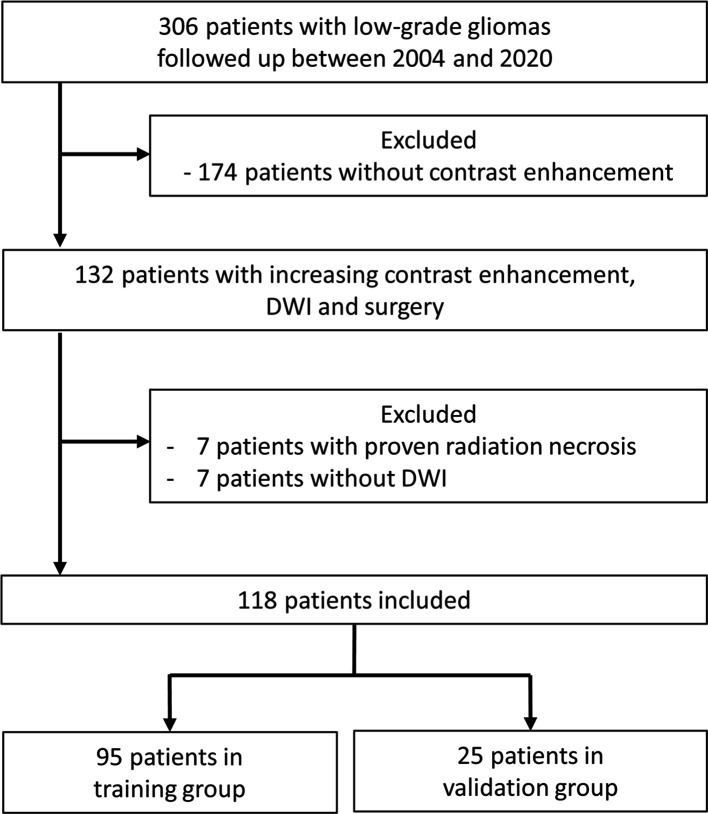
Flowchart of patient selection process. DWI, diffusion-weighted imaging.

Between 2004 and 2020, 306 patients with LGGs were regularly followed up in our institution after initial diagnosis and treatment. On follow-up MRI studies, 132 patients had 149 instances of increasing contrast enhancement leading to surgery. Seven patients with 9 instances of radiation necrosis were excluded. Eight instances of increasing contrast enhancement were excluded due to the lack of DWI studies. Removal of brain regions with increasing contrast enhancement was confirmed in 44 instances with intraoperative MRI, in 34 with postoperative MRI performed within 1 week, and in 46 with postoperative MRI performed between 2-12 weeks. Our final study population consisted of 118 patients with 132 MRI studies (48 grade II, 40 grade III, and 44 grade IV). **Figure 1** shows the patient selection process.

### Clinical and Imaging Information

Patient medical records were retrospectively reviewed to collect information including sex, age, and Karnofsky Performance Scale score (KPS) at the time of increased contrast enhancement, histologic subtypes (diffuse astrocytoma, oligoastrocytoma, or oligodendroglioma), isocitrate dehydrogenase 1 (IDH1) mutation status (12), disease duration (time interval between first histopathologic diagnosis of LGG and subsequent increased contrast enhancement), adjuvant therapy received before increased contrast enhancement, post-radiation therapy duration (time interval between the end of radiation therapy and subsequent increased contrast enhancement), and tumor grades associated with increased contrast enhancement. The first follow-up MRI studies after the last operation were reviewed for the presence of baseline residual tumor.

### MRI Parameters

MRI examinations were performed at 1.5 T (N = 14) or 3.0 T (N = 118). All examinations included a T2-weighted sequence, DWI, and T1-weighted sequences acquired before and after administration of 0.1 mmol/kg body weight gadopentetate dimeglumine (Magnevist; Schering, Berlin, Germany). MRI parameters are provided in **Table 1**. Isotropic DWI and ADC maps were generated using software provided by the manufacturers.

**Table 1:** MRI parameters.

Field strength	Vendor	Model	Patients	Sequence	TR (ms)	TE (ms)	TI (ms)	In-plane resolution (mm^2^)	Slice thickness (mm)	Slice gap (mm)
3 T	Siemens	Magnetom Trio	87							
				T2W	4000	90		0.43 × 0.43	4	0
				Post-contrast MPRAGE	2000	2.6	900	1.0 × 1.0	1	0
				DWI	5300	93		1.15 × 1.15	4	0
3 T	Philips	Ingenia	24							
				T2W	4500	100		0.68 × 0.68	4	1
				Post-contrast T1TFE	8	3.5	950	1.0 × 1.0	1	0
				DWI	4000	60		1.78 × 1.78	4	1
3 T	GE	Discovery MR750	7							
				T2W	5400	107		0.43 × 0.43	4	1
				Post-contrast BRAVO	8.2	3.2	450	1 × 1	1	0
				DWI	6000	65		0.86 × 0.86	4	1
1.5 T	Philips	Intera	6							
				T2W	4000	90		0.41 × 0.41	5	1.5
				Post-contrast T1W	420	11		0.41 x 0.41	5	1.5
				DWI	3200	60		0.82 × 0.82	5	1.5
1.5 T	GE	Optima MR450	8							
				T2W	5300	100		0.45 × 0.45	5	2
				Post-contrast BRAVO	7.8	3.2	450	1 × 1	1	0
				DWI	6000	74		0.86 × 0.86	5	2

DWI performed using 3 diffusion gradients with b values 0 and b = 1000 s/mm^2^. T2W, T2-weighted; T1W, T1-weighted; DWI, diffusion-weighted imaging; TR, repetition time; TE, echo time; TI, inversion time; MPRAGE, magnetization-prepared rapid acquisition with gradient echo; TFE, turbo field echo; BRAVO, brain volume imaging.

### Measurements of Tumor Size and ADC

All imaging data were transferred to an independent workstation and processed using nordicICE (nordic Image Control and Evaluation Version 2, Nordic Imaging Lab, Bergen, Norway). Co-registration of T2-weighted and post-contrast T1-weighted images to ADC maps were based on a 3D non-rigid transformation and mutual information. Adequacy of registration was visually assessed and manually adjusted. Blinded to the final pathologic results, 2 board-certified neuroradiologists with 20 and 17 years of experience independently measured contrast enhancement size, whole tumor size, and tumor ADC on all MRI studies. Post-contrast T1-weighted images in axial, coronal, and sagittal planes were used to localize increased contrast-enhancing tumor portions. These tumor portions were carefully chosen to include as much of the enhancing regions as possible and avoid the inclusion of necrosis, cysts, hemorrhage, edema, calcifications, and normal-appearing brain. Size of the contrast-enhancing regions was the product of the largest diameter of the increased contrast-enhancing portion and its perpendicular length on a single post-contrast transverse image. The size of the whole tumor, which included both enhancing and non-enhancing components, was measured on transverse T2-weighted images. If multiple lesions were present, the largest 3 were selected and their products were summed.

**Figure 2:**
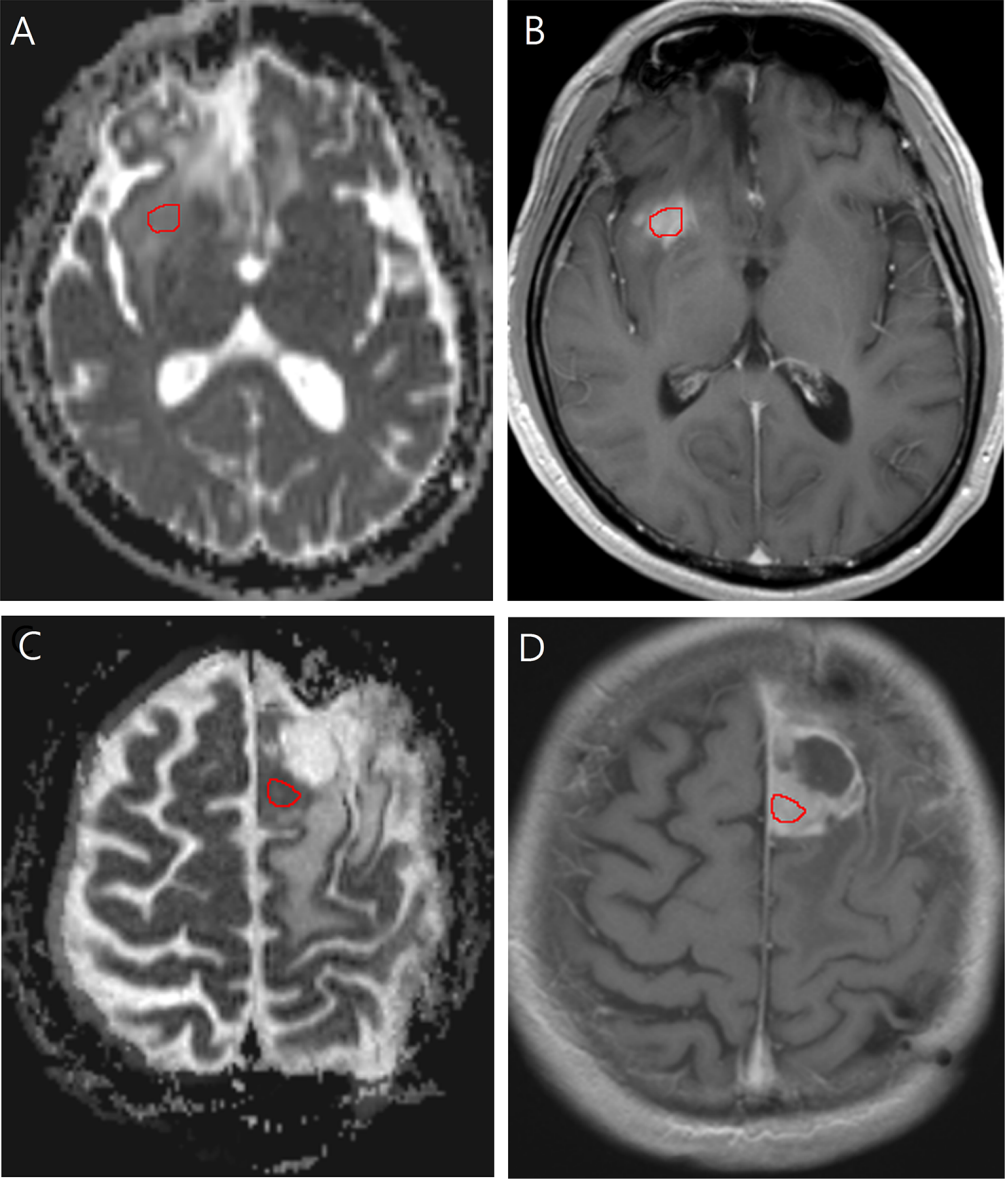
Images in a 40-year-old man with low-grade glioma with increased contrast enhancement but preserved grade II histology. **(A)** Axial apparent diffusion coefficient, and **(B)** Axial post-contrast T1-weighted images show region-of-interest placement in the contrast-enhancing solid portion of the right frontal tumor. Images in a 58-year-old man with low-grade glioma with increased contrast enhancement and malignant transformation. **(C)** Axial apparent diffusion coefficient, and **(D)** Axial post-contrast T1-weighted images show region-of-interest placement in the contrast-enhancing solid portion of the left frontal tumor.

ADC was measured by placing a region of interest (ROI) of 30 mm^2^ or larger on the tumor portion with increased contrast enhancement (**Figure 2**). The ROI was drawn to cover the largest axial tumor cross-section, after excluding necrosis, macroscopic hemorrhages, and calcifications.

### Morphologic Assessment

Blinded to final pathology results, 3 board-certified neuroradiologists with 6, 17, and 20 years of experience independently assessed the contrast enhancement patterns. Contrast enhancement patterns were categorized into solid (>70% area of the whole tumor on transverse image), scattered, or rim enhancing.

### Statistical Analysis

Intra-class correlation coefficient (ICC) analysis, with a two-way random-effects model, was used to assess agreement between observers for measurements of ADC, whole tumor size, and contrast enhancement size. For each ROI of these measurements, the mean of the observers’ measurements was adopted as the final value. Fleiss’s kappa testing was used to evaluate observer agreement for contrast enhancement patterns and the majority’s opinion was designated as the final pattern.

To validate our model for the prediction of MT in LGGs, patients were split into training and validation groups. According to the time of histopathologic diagnosis, instances diagnosed between 2004 and 2018 were included in the training group and those diagnosed between 2018 and 2020 in the validation group.

Data of the training group were used to develop the study model. Univariate logistic regression was applied to test if the following variables could predict MT: sex, age, KPS, histologic subtype, IDH1 mutation, disease duration, presence of baseline residual tumor, radiotherapy (RT), post-RT duration, chemotherapy, whole tumor size, contrast enhancement size, contrast enhancement pattern, and ADC. Selected variables with *P*-values < 0.10 by univariate analysis were subjected to multivariate analysis using logistic regression with a backward selection procedure. Starting from the highest *P*-value, a backward elimination process by using the Wald test was applied to discard variables that did not contribute significantly to the prediction concluding with the most parsimonious model to identify MT (13). Odds ratios and 95% CIs were calculated to demonstrate the relative risk of each significant factor for MT. Using receiver operating characteristic curve analysis, areas under the curve and cutoff values of statistically significant factors and regression models were determined. Cutoff values with the highest sensitivity and lowest false-positive rates were chosen to calculate sensitivity, specificity, and accuracy of each significant factor and model. A commercially available statistical software package (SPSS 23, IBM, Armonk, NY) was used for analysis, and *P*-values < 0.05 were considered to indicate significance.

By inputting the data of the validation group into the study model formula developed by multivariable regression, the MT probabilities for each instance of the validation group were obtained. Using a cut-off probability of 0.5, these MT probabilities were tabulated to calculate the respective sensitivity, specificity, PPV, and NPV for this group to validate the study model.

To further validate the study model developed using multivariate regression, we performed classification and regression trees method (CART) with k-fold cross validation using the ‘Tree’ command in the SPSS 23 software package (14, 15). All patients were randomly divided into a training set (80%) and a validation set (20%). Using data of the training set, the CART was applied to develop a model with MT as the dependent variable. The independent variables were the parameters included in the most parsimonious model generated using multivariate regression. Five-fold cross validation was performed to validate the model based on the selected parameters. This model was applied to the validation set to assess the performance of the prediction.

## Results

### Patient Demographics

**Table 2** is an overview of clinical and imaging information of 132 instances. One hundred and six instances (median age, 46 years; interquartile range, 27–65 years; 68 male patients) were included in the training group and 26 instances were included in the validation group.

**Table 2:** Clinical and imaging data of 132 instances with low-grade gliomas demonstrating increased contrast enhancement.

Clinical and Imaging Information	Training Group (N =106)	Validation Group (N = 26)	All Instances (N = 132)
Sex			
Female	38 (55.9%)	8 (30.8%)	46 (34.8%)
Male	68 (64.2%)	18 (69.2%)	86 (65.2%)
Age range (year)	48 ± 12*	51, 23^#^	47, 19^#^
Karnofsky performance status	90, 10^#^	88.2 ± 9.1*	90, 10^#^
Histologic subtype			
Diffuse astrocytoma	32 (30.2%)	11 (42.3%)	43 (32.6%)
Oligoastrocytoma	30 (28.3%)	5 (19.2%)	35 (26.5%)
Oligodendroglioma	44 (41.5%)	10 (38.5%)	54 (40.9%)
Isocitrate dehydrogenase 1 mutation			
Wild-type	14 (13.2%)	4 (15.4%)	18 (13.6%)
Mutant	66 (62.3%)	20 (76.9%)	86 (65.2%)
Not available	26 (24.5%)	2 (7.7%)	28 (21.2%)
Baseline residual tumor			
Yes	80 (75.5%)	14 (53.8%)	94 (71.2%)
No	26 (24.5%)	12 (46.2%)	38 (28.8%)
Disease duration (year)	4.6, 6^#^	4.8, 6.3^#^	4.7, 5.9^#^
Adjuvant therapy			
Radiotherapy	80 (56.3%)	22 (100%)	102 (61.4%)
Carmustine implant	49 (34.5%)	0	49 (29.5%)
Temozolomide	13 (9.2%)	0	15 (9.0%)
Post- Radiotherapy duration (month)	42.8, 68.4^#^	56.7 ± 46.1*	42.8, 60.1^#^
Whole tumor size (cm^2^)	8, 14.6^#^	9.8, 18.4^#^	9, 14.3^#^
Contrast enhancement size (cm^2^)	5, 13.5^#^	10.9 ± 12.8*	5, 13.3^#^
Contrast enhancement pattern			
Solid	32 (30.2%)	7 (26.9%)	39 (29.5%)
Scattered	44 (41.5%)	11 (42.3%)	55 (41.7%)
Rim	30 (28.3%)	8 (30.8%)	38 (28.8%)
Apparent Diffusion Coefficient (×10-6 mm2/s)			
< 968	56 (52.8%)	16 (61.5%)	72 (54.5%)
> 968	50 (47.2%)	10 (38.5%)	60 (45.5%)
Tumor grade			
II	42 (39.6%)	6 (23.1%)	48 (36.4%)
III	30 (28.3%)	10 (38.5%)	40 (30.3%)
IV	34 (32.1%)	10 (38.5%)	44 (33.3%)

*Data are mean ± SD; ^#^data are median, interquartile range.

### Interobserver Agreement

There were excellent interobserver agreements in the measurement of contrast enhancement size (ICC = 0.958, 95% CI = 0.941–0.970, *P* < 0.001), whole tumor size (ICC = 0.935, 95% CI = 0.903–0.955, *P* < 0.001) and tumor ADC (ICC = 0.924, 95% CI = 0.891–0.947, *P* < 0.001). Interobserver agreement among the 3 readers was substantial-to-perfect for categorization of contrast enhancement pattern (Fleiss’ kappa coefficient = 0.806, 95% CI = 0.801–0.810, *P* < 0.001).

### Study Model Development

**Figure 3:**
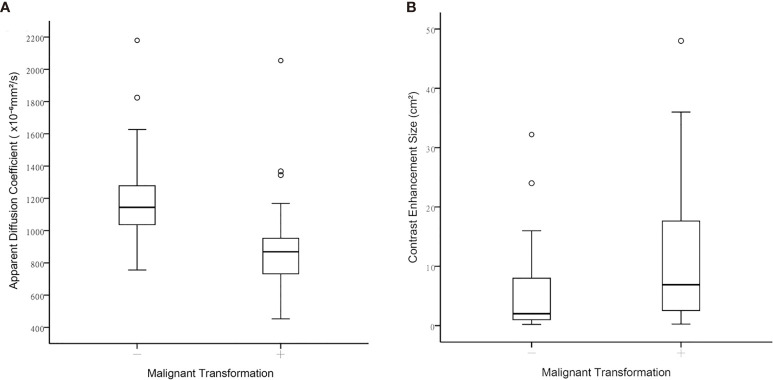
Boxplots between contrast-enhanced tumor portions with malignant transformation and those that remained WHO grade II regarding **(A)** Apparent diffusion coefficient, and **(B)** Contrast-enhancement size.

**Figure 4:**
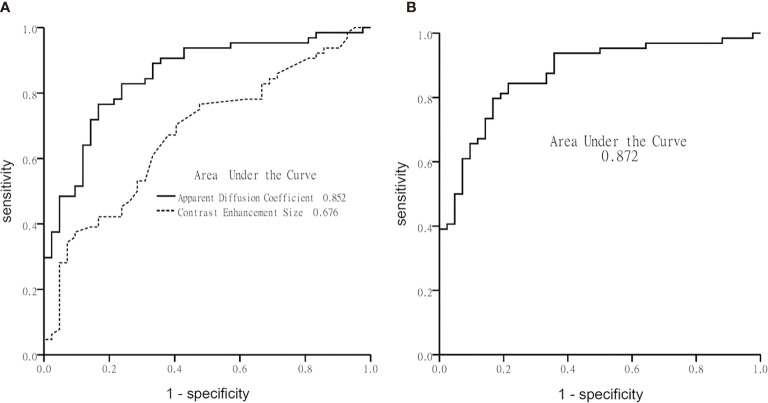
Graphs show receiver operating characteristic curve plotted using calculated sensitivity against 1-specificity to assess test performance (area-under-the-curve) in diagnosing malignant transformation. **(A)** Apparent diffusion coefficient and contrast-enhancement size, and **(B)** the multivariate logistic regression model.

**Table 3** illustrates the results of univariate analysis in which previous radiotherapy (*P* = 0.034), larger whole tumor size (*P* = 0.033), larger contrast enhancement size (*P* = 0.006), and lower ADC (*P* < 0.001) were associated with MT (**Figure 3**). On ROC analysis, the discriminative power of contrast enhancement size measured with AUC was 0.67 (95% CI: 0.57, 0.78). With 3.25 cm^2^ as the cutoff value, contrast enhancement size predicted MT with a sensitivity of 48/64 (75%), specificity of 22/42 (52%), and accuracy of 70/106 (66%) (**Figure 4**). The discriminative power of ADC measured with AUC was 0.85 (95% CI: 0.79, 0.93). With the cutoff value of 968.07 ×10^-6^ mm^2^/seconds, using ADC predicted MT with a sensitivity of 55/64 (86%), specificity of 28/42 (67%), and accuracy of 83/106 (78%) (**Figure 4**).

**Table 3:** Univariate analysis of factors associated with malignant transformation in the study group (106 instances).

Factors	Malignant Transformation		*P* Value		OR			95% CI
	No	Yes						
Sex			.70		1.18			0.52-2.64
Male	26	42						
Female	16	22						
Age (year)	50 ± 12	47 ± 13	.09		0.97			0.94-1.00
Karnofsky performance status	90, 12.6	90, 15	.52		1.01			0.98-1.05
Histologic subtype			.29		NA			NA
Diffuse astrocytoma	13	19						
Oligoastrocytoma	15	15						
Oligodendroglioma	14	30						
Isocitrate dehydrogenase 1 mutation			.38		NA			NA
Yes	25	41						
No	13	13						
Disease duration (year)	4.2, 4.0	4.9, 4.2	.59		1.00			1.00-1.00
Baseline residual tumor			.13		0.47			0.18-1.25
Yes	35	45						
No	7	19						
Radiotherapy			.03		9.46			1.18-75.78
Yes	36	44						
No	6	20						
Post-radiotherapy duration (month)	42.5, 48.4	46, 49.1	.99		0.99			0.99-1.01
Chemotherapy			.12		1.94			0.85-4.47
None	30	36						
Carmustine implant	16	33						
Temozolomide	1	12						
Whole-tumor size (cm^2^)	3.8, 9.7	12.0, 9.1	.03		1.05			1.00-1.09
Contrast enhancement size (cm^2^)	2.0, 7.0	6.8, 10.2	.01		1.09			1.02-1.15
Contrast enhancement pattern (cm^2^)			.64		0.89			0.53-1.48
Solid	11	21						
Scattered	19	25						
Rim	12	18						
Apparent Diffusion coefficient (×10^-6^ mm^2^/s)	1137.0 ± 287.8	880.3 ± 257.2	< 0.001		0.99			0.99-1.00

Data are mean ± SD for age; data are median, interquartile range for Karnofsky performance status, disease duration, post-radiotherapy duration, whole-tumor size, contrast enhancement size, and apparent diffusion coefficient.

Using multivariate logistic regression analysis with a backward selection procedure, the most parsimonious model for predicting MT was developed and consisted of age in years (P = 0.110), radiotherapy (*P* = 0.168), contrast enhancement size in cm^2^ (*P* = 0.015), and ADC in mm^2^/s (*P* < 0.001). The model formula was logit (probability) = 8.152 - 0.038*Age -0.853*Radiotherapy + 0.081*Contrast enhancement size - 0.006*ADC. On ROC analysis, the AUC of this model was 0.87 (95% CI: 0.81, 0.94) (**Figure 4**). With 0.5 as the probability cutoff value, the sensitivity, specificity, and accuracy of this model in diagnosing MT were 56/64 (88%), 33/42 (79%), and 89/106 (84%), respectively (**Table 4**).

**Table 4:** Diagnostic accuracy of malignant transformation for study model and model validation.

Statistical Algorithm		Sensitivity	Specificity	PPV	NPV	Accuracy
Multivariate regression	Study model (106 instances)	0.88	0.79	0.86	0.80	0.84
	Model validation (26 instances)	0.85	0.83	0.94	0.63	0.85
						
Classification and regression trees with 5-fold cross validation	Study model (106 instances)	0.88	0.83	0.89	0.80	0.86
	Model validation (26 instances)	0.89	0.88	0.94	0.78	0.88

### Study Model Validation

By inputting the data of the validation group (26 instances) into the study model formula, the model correctly classified MT in 22 of 26 instances (85%), with a sensitivity of 17/20 (85%) and specificity of 5/6 (83%) (**Table 4**).

Using the CART with a 5-fold cross validation and incorporating age, radiotherapy, contrast enhancement size, and ADC as the independent variables, the model generated with the training set (106 instances) correctly classified MT in 91 of 106 instances (86%), with a sensitivity of 58/66 (88%) and specificity of 33/40 (83%) (**Table 4**). By applying this model to the validation set (26 instances), MT was correctly classified in 23 of 26 instances (88%), with a sensitivity of 16/18 (89%) and specificity of 7/8 (88%) (**Table 4**).

## Discussion

Our results show that if we used increasing contrast enhancement as an indication of MT in LGGs, one-third (48/132) of them would remain grade II. A multivariate logistic regression model, including age, presence of radiotherapy, ADC and contrast enhancement size, was established to predict MT (accuracy: 84%, sensitivity: 86%, specificity: 79%). This model was further validated by using data of 26 recently recruited instances (accuracy: 85%, sensitivity: 85%, specificity: 83%). By inputting clinical information and common MRI parameters into the model formula the probability of MT was predicted with high accuracy.

In high-grade gliomas, decreased ADC (16, 17) is associated with increased tumor cellularity. MT is expected to show decreased ADC due to increased cellularity. Previous reports using ADC for differentiation among glioma grades show variable results. Differentiation between LGGs and high-grade gliomas can be achieved using DWI in which ADC values of LGGs are significantly higher than those of high-grade tumors (18, 19). A recent report revealed significant ADC differences between grades II and III, grades II and IV, and between grades II and III-IV gliomas (9). Conversely, a study of non-enhancing gliomas found no significant ADC differences between LGGs and high-grade gliomas (20), Moreover, a considerable overlap of ADC values have been found between grade II and grade IV gliomas (21) and between grade III and grade IV gliomas (19). These inconsistencies may be partly explained by tissue heterogeneity in glial tumors (22). Because tumor grading depends on the location of biopsies or surgical resection, such heterogeneity may cause sampling errors and thus inaccurate grading. In these previous studies, the location of contrast enhancement was not cross-referenced to that of the ROI (8, 9, 18, 19). ADC values have been used to detect early MT in LGG. In a study of 18 patients undergoing MT (10), low intensity on DWI was used to target possible MT. Similarly, because of heterogeneity of high-grade glioma (23) and MT (11), portions of tumors with varying ADC values may coexist making it difficult to locate those with the lowest diffusion further reducing reproducibility. Of note is that in one series contrast enhancement occurred simultaneously with restricted diffusion in 12/18 (66%) patients and appeared about eight months later in the remaining 6/18 (33%) patients with restricted diffusion (10). Contrast enhancement was also found in the location of restricted diffusion about 3 months later in 23/27 (85%) patients with glioblastoma (10). These results suggest that contrast enhancement is likely to appear within months after restricted diffusion in high-grade gliomas. In our study, ADC was only measured in tumor portions with increasing contrast enhancement. Given that our model accurately diagnosed MT in a subset of patients with contrast-enhancing tumors, the combined use of ADC measurements and increasing contrast enhancement improved the specificity by limiting the ADC measurement to the contrast-enhancing tumor portions thus counteracting the effect of tumor heterogeneity.

Recently, an MRS study diagnosing MT of LGGs with increased contrast enhancement showed an accuracy of 89.6% (4). In that study, using single-voxel proton MRS, the NAA/Cho ratio was the only significant factor diagnostic of MT. Unlike that study, ours used DWI and contrast-enhanced imaging which are included in routine MRI protocols. Moreover, pre-localization of lesion as needed for MRS was not required and imaging data can be retrospectively processed to evaluate different tumor components, for example, non-enhancing components. However, when the results of imaging are uncertain using conventional MRI techniques, MRS can be obtained to improve one’s interpretation.

In our study, the median time to MT was 5 years and thus comparable to previously reported times to MT that range from 2.7 to 5.4 years (24, 25). However, in our study, none of the previously identified factors (24–26), including old age, male sex, multiple tumor locations, tumor size > 5 cm, adjuvant temozolomide, presence of residual tumor, astrocytoma histology, and IDH wild-type, were significant predictors of MT. This discrepancy may be attributed to different criteria used for MT, such as the fact that in our study MT was confirmed with histology but in others, this confirmation was imaging-based in some patients. While most of the previously mentioned factors were analyzed in our study, ADC was not assessed or analyzed in those other studies. In our study, the combined effect of ADC and contrast enhancement size on MT was stronger than the effect of other factors thus diluting the effect of those factors in the multivariate regression model. More importantly, in other studies, the factors were assessed for associations with MT, but in ours, various factors were used to develop a model to predict MT.

Deep learning has recently become a dominant form of supervised machine learning method that uses a network architecture for a specific application (27). For classification in neuroimaging, imaging features are extracted and act as inputs to enter a neural network, like the convolutional neural network (CNN), which outputs a probability of the image belonging to each class. Deep learning methods have been applied in multiple aspects of gliomas, using MRI metrics to predict long-term outcome, treatment response like pseudopregression, and tumor genetics including 1p19q codeletion, O-6-methylguanine DNA-methyltransferase promoter, and IDH mutations (28). A deep learning approach would be able to model more complex and non-linear relationships between dependent and independent variables. In contrast, the model presented in our study is a simple linear relationship of limited clinicoradiologic features. Our model may not be as robust as one built with deep learning in the prediction of MT, but it allows assessments of the individual associations between MT and clinicoradiologic parameters. This information could be clinically important and is not available with deep learning method. However, if a larger sample size was available, applying a radiomic approach with deep learning to predict MT of gliomas would be a focus of further investigation.

Only LGGs having increasing contrast enhancement were included in our study. The exclusion of non-enhancing tumors was a limitation of our study. Further study of MT in non-enhancing tumors is necessary and should be performed using an alternative approach instead of manual ROI placement, such as whole-tumor histogram analysis. Second, ADC measurements may be affected by heterogeneity in MRI units and protocols. Unfortunately, these heterogeneities are inevitable in a study of patients imaged over 17 years although it has been reported that variability of ADC values across platforms is small (29). Thirdly, the use of 2-dimensional measurements was a limitation of our study. A recent study comparing volumetric segmentation and bidimensional products in the assessment of glioblastoma progression revealed that using the bidimensional measurement was approximately 30% less accurate and tended to underestimate tumor progression (30). Lastly, the inclusion of patients from a single institution may limit the generalizability of our findings, which can be improved by performing a more comprehensive multicenter study using different MRI scanners and MRI protocols.

In conclusion, a model incorporating ADC and contrast enhancement size was established to predict MT in low-grade gliomas with increased contrast enhancement. Compared with using contrast enhancement size alone, taking into consideration ADC more accurately diagnoses MT of low-grade gliomas.

## Data Availability Statement

The raw data supporting the conclusions of this article will be made available by the authors, without undue reservation.

## Ethics Statement

The studies involving human participants were reviewed and approved by Chang Gung Medical Foundation Institutional Review Board. Written informed consent for participation was not provided by the participants’ legal guardians/next of kin because: This retrospective study was performed after institutional review board approval. The need to obtain patient informed consent was waived by our review board (202100387B0).

## Author Contributions

AW and CT primarily designed the study. TS, K-CW, P-YC, and MC provided suggestions for improvements from technical and medical perspective. AW, CT, and TS collected and analyzed data. All co-authors provided feedback and were involved in manuscript revision. All authors contributed to the article and approved the submitted version.

## Funding

This work was funded by grants from the Ministry of Science and Technology, Taiwan (MOST 108-2314-B-182A-015, MOST 108-2314-B-182A-016, MOST 108-2314-B-182A-044).

## Acknowledgments

The authors thank Mr. Morris MF Wu for providing advice on statistical analysis.

## Conflict of Interest

The authors declare that the research was conducted in the absence of any commercial or financial relationships that could be construed as a potential conflict of interest.

## Publisher’s Note

All claims expressed in this article are solely those of the authors and do not necessarily represent those of their affiliated organizations, or those of the publisher, the editors and the reviewers. Any product that may be evaluated in this article, or claim that may be made by its manufacturer, is not guaranteed or endorsed by the publisher.
